# Supplementary material for: Novel scFv against Notch Ligand JAG1 Suitable for Development of Cell Therapies toward JAG1-Positive Tumors
Source: Biomolecules. 2023 Mar 2;13(3):459. doi: 10.3390/biom13030459 (PMC10046313; doi:10.3390/biom13030459)
Supplement: Supplementary file 1 [file biomolecules-13-00459-s001.zip › biomolecules-2185606-supplementary.pdf]

Supplementary Materials: Novel scFv against the Notch ligand JAG1 suitable for the development of cell therapies towards JAG1-positive tumors

Gabriela Silva, Ana F. Rodrigues, Susana Ferreira, Carolina Matos, Rute P. Eleutério, Gonçalo Marques, Khrystyna Kucheryava, Ana R. Lemos, Pedro M. F. Sousa, Rute Castro, Ana Barbas, Daniel Simão, Paula M. Alves

Methods

Cell lines and reagents

MCF-7 (ATCC, Manassas, VA, USA, HTB-22) and MDA-MB-231 (ATCC, HTB-26) cells were cultured as described in [1]. SKBR3 cells (HTB-30) were cultured in McCoy's medium (Sigma-Aldrich, M9309) whereas BT474 (HTB-20), HCC1187 (CRL-2322), HCC1806 (CRL-2335), HCC1954 (CRL-2338), MDA-MB-468 (HTB-132), NCI-N87 (CRL-5822), NCI-H1573 (CRL-5877), Raji (CCL-86) (all from ATCC) and U-251-MG (ECACC, #09063001) cells were cultured in RPMI 1640 medium (Gibco, #61870) supplemented with 10% (v/v) heat-inactivated fetal bovine serum (FBS, #10270-106) and 100 μg/mL penicillin and streptomycin (#15140-122). HPAC (ATCC, CRL-2119) cells were cultured in DMEM:F12 (ATCC, #30-2006) supplemented with 5% (v/v) FBS, 40 ng/mL hydrocortisone (H088-1G), 0.002 mg/mL insulin (I0516) (all from Sigma-Aldrich), 0.005 mg/mL transferrin (Lonza, CC-4205) and 0.1 mg/mL rhEGF (StemCell, #78006.1). All cells were cultured at 37 ºC in a humidified atmosphere with 5% (v/v) CO_2 according to the supplier's instructions and tested for the absence of mycoplasma.

Surface Plasmon Resonance (SPR assays)

The interaction kinetics of anti-JAG1 antibodies (Abs) towards rhJAG-1-ECD was evaluated using a Biacore 4000 instrument (Cytiva). rhJAG-1-ECD was immobilized at 1 μg/mL in 10 mM Sodium Acetate pH 5.5. HBS-P+, consisting of 10 mM HEPES pH 7.4, 150 mM NaCl and 0.5% v/v Surfactant P20, was used as background buffer during immobilization. Prior to immobilization, the carboxymethylated surface of the chip was activated with a 1:1 ratio of 400 mM 1-ethyl-3-(3-dimethylaminopropyl)-carbodiimide and 100 mM N-hydroxysuccinimide for 10 min. Each anti-JAG1 Ab was coupled to the surface with a 1 to 4 min injection time at a flow rate of 10 μL/min to reach 20 to 80 RU. The remaining activated carboxymethylated groups were blocked with a 7 min injection of 1 M ethanolamine pH 8.5. Abs were directly diluted in running buffer (HBS-P+) and injected at 10 different concentrations using 2-fold dilution series starting from 0.2 μM based on the previously obtained ELISA EC_{50} values. Interaction analysis cycles consisted of a 220-second sample injection (30 μL/min; association phase) followed by 600-second of buffer flow (dissociation phase). All sensorgrams were processed by first subtracting the binding response recorded from the control surface (reference spot), followed by subtracting of the buffer blank injection from the reaction spot. All datasets were fit to a simple 1:1 Langmuir interaction model to determine kinetic rate constants. Experiments were performed on a Biacore 4000 (Cytiva, Uppsala, Sweden) at 25 °C and the interactions were evaluated using the provided Biacore 4000 evaluation software.

CAR lentiviral preparations

Lentiviral vectors were produced and titrated in HEK293T cells. For production, HEK293T cells were seeded in DMEM 10% (v/v) FBS at a concentration of 0.15 x 10^6 cells/cm^2 and transfected at the moment of seeding – transfection at seeding protocol – with a total of 3.3 μg of plasmid DNA per million cells with the following proportion:

0.7 μg of pMDLg/pRRE, 0.2 μg of pRSV-Rev, 0.6 μg of envelope (VSV-G) and 1.8 μg of vector transgene/CAR construct. Lentiviral packaging [2] and envelope plasmids were kindly provided by Didier Trono through Addgene plasmid repository (plasmids #12251, #12253, #12259). Lentiviral packaging [2] and VGS-G envelope plasmids were kindly provided by Didier Trono through Addgene plasmid repository (plasmids #12251, #12253, #12259). Polyethylenimine (PEI, Polysciences Inc, Hirschberg an der Bergstrasse, Germany) was used as transfection reagent at a mass ratio of 1:1.5 (DNA:PEI). At 24 h post-transfection, the culture medium was exchanged by RPMI 10% (v/v) FBS. At 48 h post-transfection, the supernatant containing the lentiviral particles was harvested, clarified through a 0.45 μm pore-size cellulose acetate filter, aliquoted and stored at -80º C until further use. Transfection efficiency was accessed at the time of harvesting by measuring the percentage of GFP^+ cells by flow cytometry using Gallios flow cytometer (Beckman Coulter, Brea, CA, USA).

Supernatants containing lentiviral particles were quantified for transducing units titter using a vector copy number (VCN) titration protocol. This method determines the number of integrated lentiviral genomes by quantitative PCR (qPCR) in lysates of transduced cells, using the long terminal repeat (LTR) of lentiviral vector as target sequence and the human albumin (ALB) as internal reference gene. Briefly, HEK293T cells were seeded in 24-well plates at 0.08×10^6 cells/cm^2 and transduced 24 h after seeding with several dilutions of lentiviral supernatant in DMEM with 10% (v/v) FBS containing polybrene (Sigma, H9268), at a final concentration of 8 μg/mL. At 48 h post-transduction, culture medium was removed, wells were washed with PBS and 100 μL of lysis buffer (#LV961A-BUFR, Global UltraRapid Lentiviral Titering Kit, System Bioscience, Palo Alto, CA, USA) was added to each cell well, followed by freezing at -80º C for 30 min. After thawing, lysates were heated at 95º C for 10 min and then centrifuged (1200 x *g*, 5 min) for clarification. Extracts were aliquoted and stored at -20º C until further use.

The detection of integrated lentiviral genomes and reference gene was done by multiplexed qPCR using LightCycler 480 Probes Master mix in a LightCycler 480 Instrument II (Roche Applied Science). Primers and probe sequences are listed in Supplementary Table S3.

Calibration curves were established with 10-fold serial dilutions of the plasmid pRRLSIN.cPPT.PGK-GFP.WPRE_ALB, which contains the lentiviral LTR and the complete cDNA of ALB gene, obtained from DNASU plasmid repository (clone HsCD00043431, Biodesign Institute, Arizona State University, Tempe, AZ, USA). VCN was determined by dividing the number of integrated lentiviral sequences to the number of ALB (reference), using the following equation:

$VCN = (copies/\mu L\ LTR\ sequence)/(copies/\mu L\ ALB\ sequence) \times N$

where "N" indicates the number of genomic copies of the reference gene per cell genome which was assumed to be 2. Lentiviral titers, in transducing units per mL (T.U./mL), were determined using the following equation:

$(T.U.)/mM = (VCN \times C \times D)/V$

where C, D and V indicate the number of cells at infection, the LV supernatant dilution and the volume of lentiviral supernatant (in mL) used for transduction, respectively

Determination of Jurkat T cell activation by anti-CD19 CARs

Non-transduced/parental and Jurkat cells transduced with each anti-CD19 CAR or Mock (7.5×10^4 in 150μL media in 96-well plates) were cultured alone or with the CD19-positive cell line Raji, pre-labelled with celltrace violet (Invitrogen, #C34571) according to the manufacturer's instructions, at 1:1 and 10:1 effector (Jurkat)/target (Raji) (E:T) ratios. After 21 h, cells were collected for staining with CD69-APC. For flow cytometric analysis, Jurkat cells were gated on celltrace violet-negative populations and cells transduced with Mock or CARs were then gated on the GFP-positive signals.

References

1. Sales-Dias, J.; Silva, G.; Lamy, M.; Ferreira, A.; Barbas, A. The Notch ligand DLL1 exerts carcinogenic features in human breast cancer cells. *PloS one* **2019**, *14*, e0217002, doi:10.1371/journal.pone.0217002.
2. Dull, T.; Zufferey, R.; Kelly, M.; Mandel, R.J.; Nguyen, M.; Trono, D.; Naldini, L. A third-generation lentivirus vector with a conditional packaging system. *J Virol* **1998**, *72*, 8463-8471, doi:10.1128/JVI.72.11.8463-8471.1998.

Supplementary Figures and Tables

Fig. S1

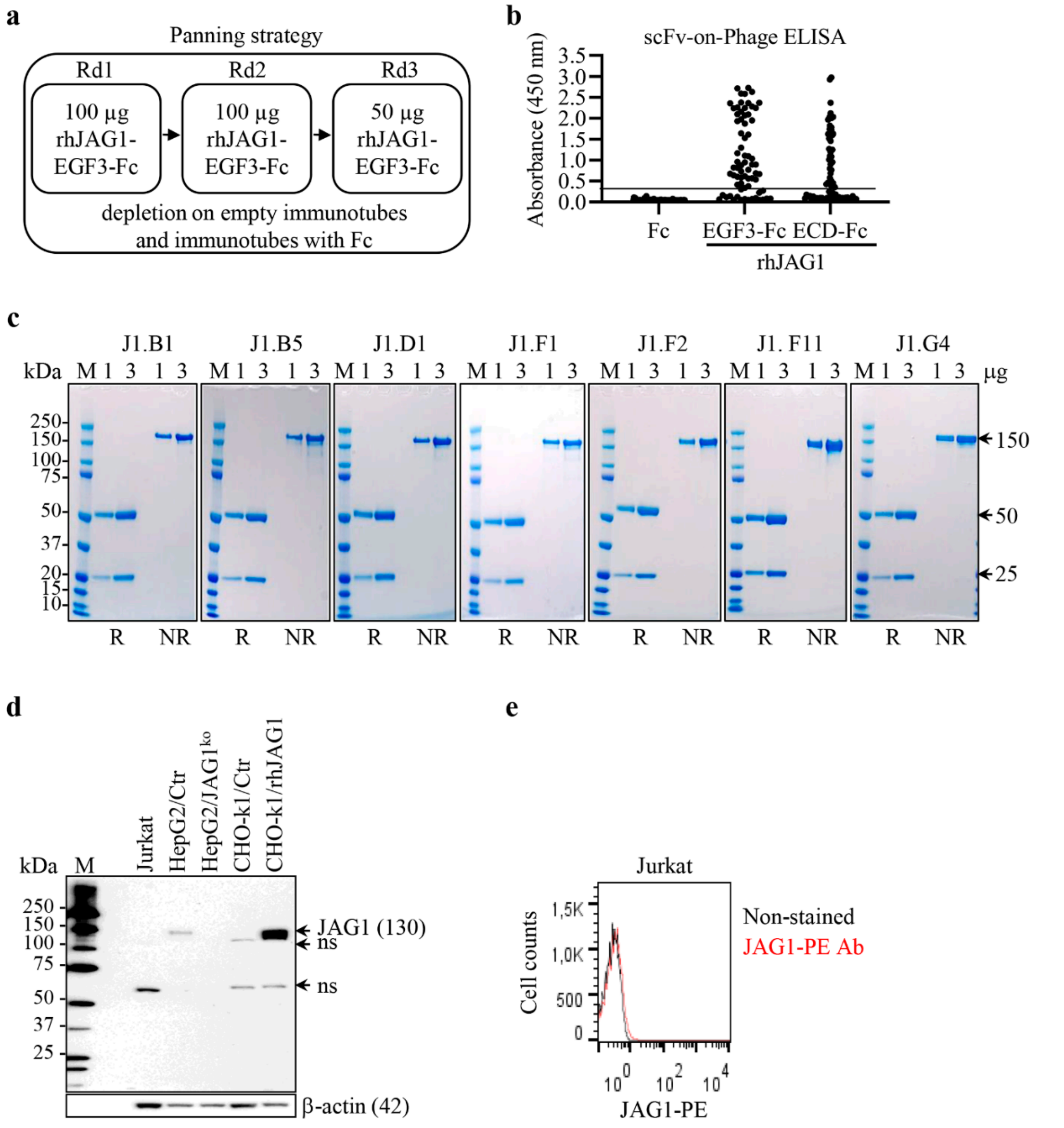

Figure S1. Selection of scFv against JAG1 and characterization of anti-JAG1 Abs. (a-b) Selection of specific anti-JAG1 scFv by phage display. **(a)** Panning strategy used to select specific scFvs with potential to block JAG1 binding to Notch receptor consisted in three selection rounds with recombinant human JAG1 protein comprising the Notch binding regions (rhJAG1-EGF3-Fc) preceded by two depletion steps in empty immunotubes and immunotubes coated with Fc control protein. **(b)** scFv-on-phage ELISA screening for the identification of selected scFvs able to bind rhJAG1-EGF3-Fc and rhJAG1-ECD (with complete extracellular domain). Clones not binding Fc control protein and presenting an absorbance value > 0.3 were considered positive. Sequencing analysis of scFvs able to bind rhJAG1 proteins allowed the identification of seven unique sequences without glycosites in their complementarity-determining regions which were reformatted into IgG1 molecules. **(c)** SDS-PAGE analysis of the indicated amounts of the seven anti-JAG1 Abs, produced in HEK293-E6 cells and purified by affinity and size exclusion chromatography in reduced (R) and non-reduced (NR) conditions. The arrows indicate protein bands with the expected molecular masses. M, precision plus protein all blue standards (BioRad, #161-0373). **(d)** Western blot analysis of JAG1 (130 kDa) expression in total protein extracts from the indicated cells. Detection of β-actin was used as loading control. ns, non-specific band. M, precision plus unstained standards (BioRad, #161-00363) after detection with precision protein streptactin-HRP-conjugate (BioRad, #1610381). **(e)** Flow cytometry analysis of JAG1 expression in Jurkat T cells.

Fig. S2

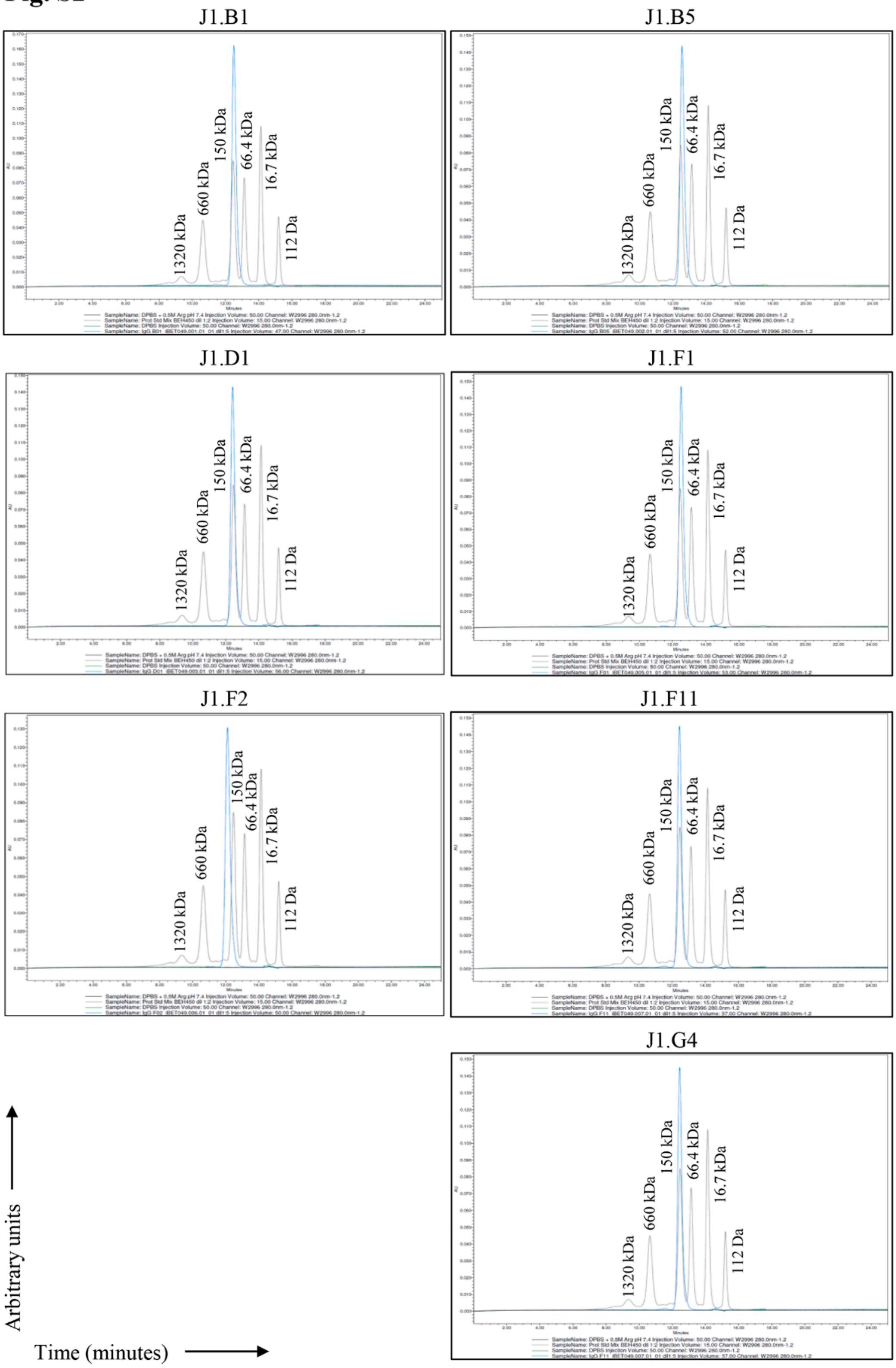

Figure S2. Size-exclusion-high-performance liquid chromatography analysis of purified anti-JAG1 Abs injected on Xbridge BEH450 column. Size-exclusion chromatogram of anti-JAG1 Abs (blue pics) compared to protein standards. Protein standard mix was injected under the same conditions, and the respective molecular weight is shown in grey lines. Protein standards used: thyroglobulin dimer (1320 kDa), thyroglobulin (660 kDa), IgG (150 kDa), BSA (66.4 kDa), myoglobin (16.7 kDa) and Uracil (112 Da). The histogram corresponding to each JAG1 Ab is shown in blue.

Fig. S3

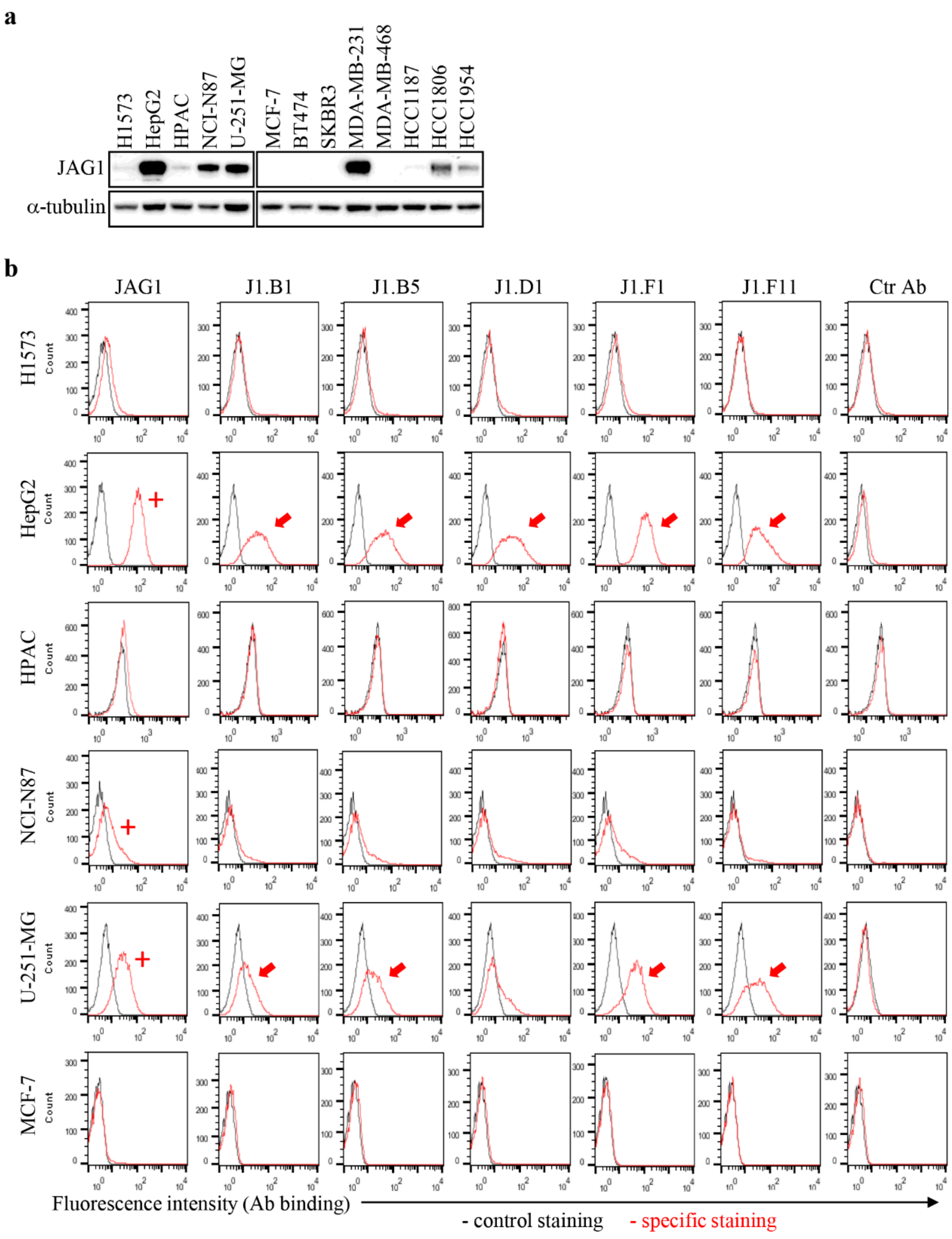
a
H1573
HepG2
HPAC
NCI-N87
U-251-MG
MCF-7
BT474
SKBR3
MDA-MB-231
MDA-MB-468
HCC1187
HCC1806
HCC1954
JAG1
α-tubulin
b
JAG1
J1.B1
J1.B5
J1.D1
J1.F1
J1.F11
Ctr Ab
H1573
HepG2
HPAC
NCI-N87
U-251-MG
MCF-7
Count
Fluorescence intensity (Ab binding)
- control staining
- specific staining

Fig. S3 continued

b

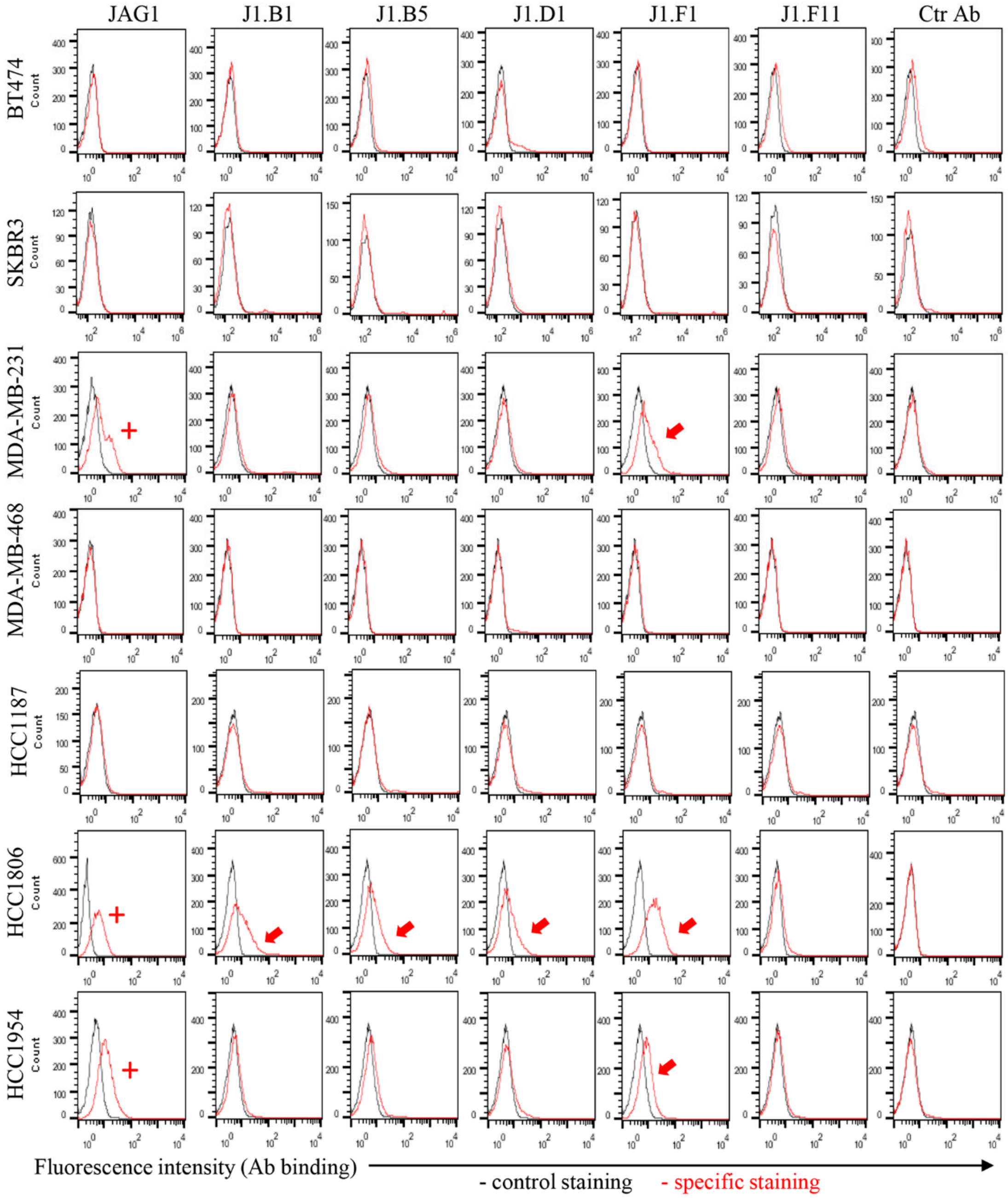

Figure S3. Binding of anti-JAG1 Abs to cellular JAG1. **(a)** Expression of JAG1 in whole cell lysates from cancer cell lines from various breast cancer subtypes (luminal A (MCF-7), luminal B (BT474), HER2+ (SKBR3) and triple-negative (MDA-MB-231/468, HCC1187/1806/1954)) and cell lines representative of tumours where JAG1 is oncogenic (lung cancer (H1573), liver cancer (HepG2), pancreatic cancer (HPAC), stomach cancer (NCI-N87), and brain cancer (U-251-MG)). Detection of α-tubulin was used as loading control. **(b)** Flow cytometry analysis of cell surface JAG1 expression (left panels) and binding of 2700 nM of each indicated anti-JAG1 Ab or Ctr Ab to the cell lines in (a). Specific staining indicates cells stained with the primary Abs. Control staining denotes unstained cells in left panels and cells stained with secondary anti-human IgG (H+L)-A488 in the other panels. The arrows indicate binding of tested anti-JAG1 Abs and + denotes JAG1 cell surface expression, determined with anti-human JAG1-PE Ab (BD Pharmigen, #565495). MDA-MB-231 cells present very low levels of surface JAG1 surface in contrast to immunoblotting data showing high JAG1 levels in whole cell lysates.

Fig. S4

```
                                  CDR1-VH                         CDR2-VH
J1.B5   EVQLLESGGGLVQPGGSLRLSCAASGFTFSSYAMSWVRQAPGKGLEWVSTIAGSGTATDY 60
J1.F1   EVQLLESGGGLVQPGGSLRLSCAASGFTFSSYAMSWVRQAPGKGLEWVSTISTSGDYTTY 60
        ***************************************************: **   * *

                                            CDR3-VH
J1.B5   ADSVKGRFTISRDNSKNTLYLQMNSLRAEDTAVYYCAKDTYAFDYWGQGTLVTVSSGGGG 120
J1.F1   ADSVKGRFTISRDNSKNTLYLQMNSLRAEDTAVYYCAKSTAYFDYWGQGTLVTVSSGGGG 120
        **************************************.*   *****************

        GS linker                           CDR1-VL
J1.B5   SGGGGSGGGGSTDIQMTQSPSSLSASVGDRVTITCRASQSISSYLNWYQQKPGKAPKLLI 180
J1.F1   SGGGGSGGGGSTDIQMTQSPSSLSASVGDRVTITCRASQSISSYLNWYQQKPGKAPKLLI 180
        ************************************************************

         CDR2-VL                                   CDR3-VL
J1.B5   YAASYLQSGVPSRFSGSGSGTDFTLTISSLQPEDFATYYCQQAYSAPSTFGQGTKVEIK  239
J1.F1   YAASALQSGVPSRFSGSGSGTDFTLTISSLQPEDFATYYCQQAYYDPTTFGQGTKVEIK  239
        **** *********************************** ****   *:***********
```

Figure S4. Sequence alignment of amino acid sequences of scFvs from lead specific anti-JAG1 Ab J1.B5 and J1.F1. Alignments were performed using the Clustal Omega multiple sequence alignment program (ttps://www.ebi.ac.uk/Tools/msa/clustalo). The respective assigned complementarity-determining regions (CDR) from the variable heavy (VH) and variable light (VL) regions and (GGGGS)3 linkers are indicated.

Fig. S5

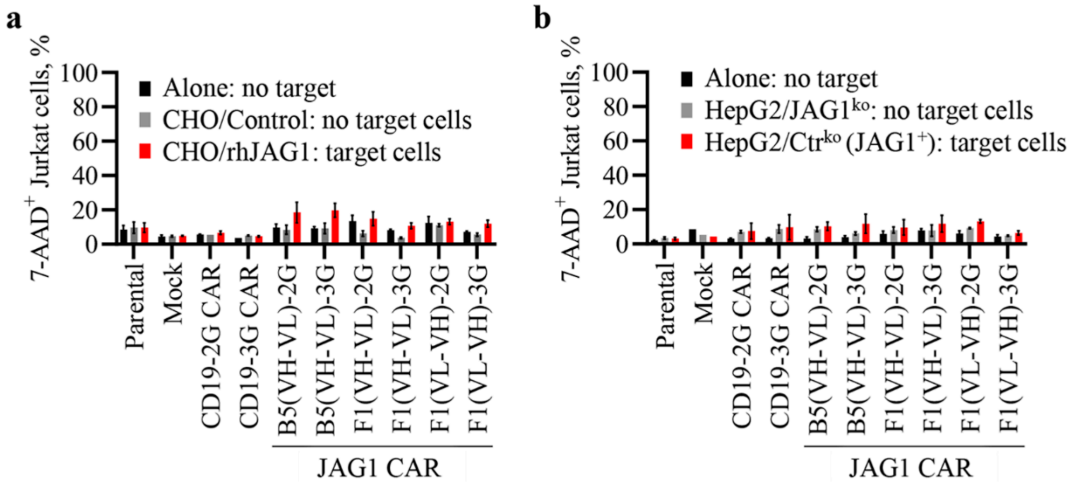

Figure S5. Effect of anti-JAG1 CARs on the viability of Jurkat T cells. **(a-b)** Jurkat parental cells and Zeocin-selected cells after transduction with Mock, anti-JAG1 CARs or anti-CD19 CARs were cultured alone or with the JAG1-negative cells CHO-k1/Control or HepG2/JAG1^{ko} (no target cells) or the JAG1-positive cells CHO-k1/rhJAG1 or HepG2/Ctr^{ko} (target cells) at the indicated Jurkat-to-target (E:T) ratios for 21 h. Then, Jurkat cells were collected, stained with 7-AAD and viability assessed by flow cytometry. The graphs show percentage of death cells (mean ± SEM) of three independent experiments performed with CHO-k1 **(a)** and four independent assays with HepG2 cells **(b)**, each condition done at least in duplicate. Anti-JAG1 CARs have no significant effect in the viability of Jurkat T cells either grown alone or in the presence of no target or target cells as compared to control parental cells, cells transduced with Mock or anti-CD19 CARs.

Fig. S6

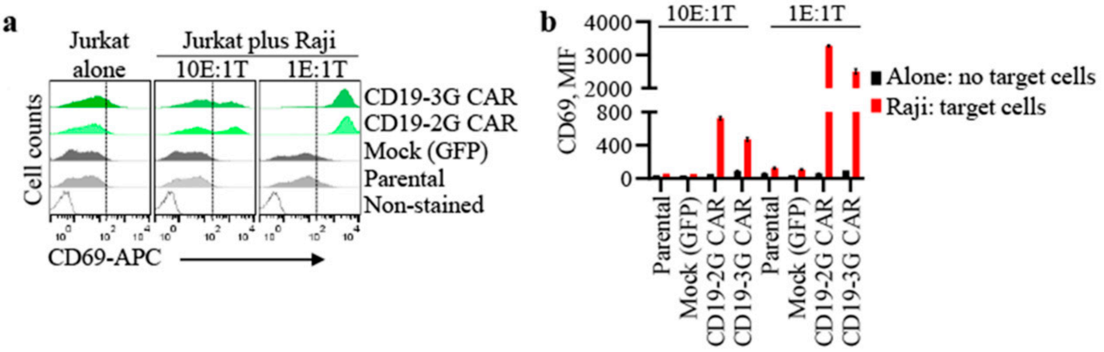

Figure S6. Activation of anti-CD19 CAR Jurkat T cells by CD19-expressing Raji cells. **(a-b)** Jurkat parental cells and Zeocin-selected cells after transduction with Mock or anti-CD19 CARs were cultured alone or with CD19-positive Raji cells at a 10:1 or 1:1 E:T ratio for 21 h. Then, Jurkat cell activation was assessed by CD69 expression in flow cytometry assays. **(a)** Representative flow cytometry plots of CD69 expression in Jurkat cells monocultures or cocultures with Raji cells from two independent experiments, each done in duplicate. Horizontal dashed lines mark CD69 induction from baseline in parental cells. (**b**) The graph shows quantification of CD69 expression (mean ± SEM) from these experiments. MIF, mean fluorescence intensity.

Fig. S7

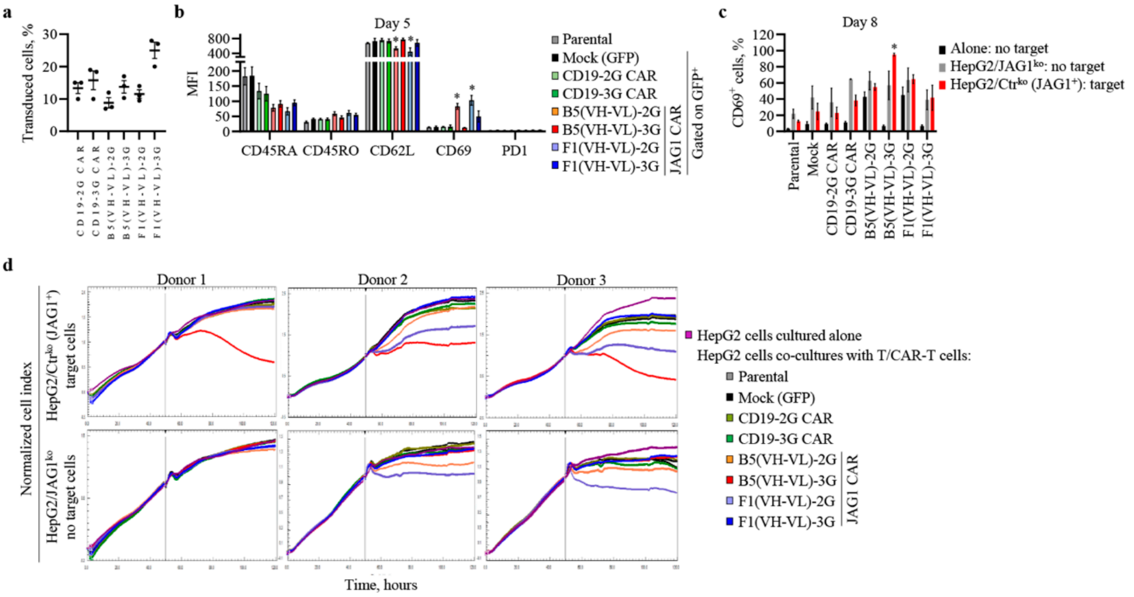

Figure S7. Phenotype and killing activity of CAR-T cells. **(a)** Percentage of transduced primary T cells from three different donors with each indicated CAR, defined as GFP-positive cells by flow cytometry. **(b)** Expression of the indicated T cell phenotype markers in non-transduced parental cells and gated GFP-positive Mock or CAR-T cells (mean ± SEM) from the three donors at day 5 post-transduction. MIF, mean fluorescence intensity. *, $P < 0.05$ vs. parental cells. **(c)** Mean percentage (± SEM) of CD69-positive cells in parental T cells or gated GFP-positive Mock or CAR-T cells from the three donors cultured alone or with JAG1-negative (HepG2/JAG1^{ko}) or JAG1-positive (HepG2/Ctr^{ko} (JAG1^{+})) cells during 21 h. *, represents significant differences ($P < 0.05$) between the indicated CAR-T cells and parental cells cultured alone. Data in (b, c) were analyzed by 2-way ANOVA with Tukey´s multiple comparisons test. **(d)** Normalized cell index of JAG1-positive (HepG2/Ctr^{ko} (JAG1^{+})) (upper panels) and JAG1-negative (HepG2/JAG1^{ko}) (lower panels) cells incubated without or with primary T cells or CAR-T cells from the three donors, as indicated, obtained in the RTCA assays. Data was acquired using the RTCA xCELLigence system.

Table S1. Molecular detail of CAR molecules.

Signal peptide	Hinge	TM domain	Co-stimulatory domain	scFv	Chain order	Linker
GMCSF	CD8A	CD8A	4-1BB	anti-CD19 from FMC63	VL-VH	218
GMCSF	CD8A	CD8A	4-1BB	anti-JAG1 from F1	VL-VH	218
GMCSF	CD8A	CD8A	4-1BB	anti-JAG1 from F1	VH-VL	$(GGGGS)_3$
GMCSF	CD8A	CD8A	4-1BB	anti-JAG1 from B5	VH-VL	$(GGGGS)_3$
GMCSF	CD8A	CD8A	CD28 and 4-1BB	anti-CD19 from FMC63	VL-VH	218
GMCSF	CD8A	CD8A	CD28 and 4-1BB	anti-JAG1 from F1	VL-VH	218
GMCSF	CD8A	CD8A	CD28 and 4-1BB	anti-JAG1 from F1	VH-VL	$(GGGGS)_3$
GMCSF	CD8A	CD8A	CD28 and 4-1BB	anti-JAG1 from B5	VH-VL	$(GGGGS)_3$

Table S2. Antibodies used in Western blot (WB) and flow cytometry (FC) assays.

Target	Host	Clone	Application	Concentration/ dilution	Conjugation	Supplier (Reference)
α-tubulin	Mouse	DM1A	WB	1:10000	-	Sigma (T6199)
Anti-human IgG (H+L) secondary antibody	Goat	Polyclonal	FC	1:1000	488	Invitrogen (A-11013)
Anti-human IgG-Peroxidase	Goat	Polyclonal	WB	1:7500	HRP	Sigma (A5420)
Anti-human IgG-Peroxidase	Mouse	Polyclonal	WB	1:10000	HRP	Sigma (A9309)
β-actin	Mouse	AC-74	WB	1:10000	-	Sigma (A5316)
CD19	Mouse	HIB19	FC	1:5	PE	BD Pharmingen (555413)
CD247 (CD3ξ)	Mouse	8D3	WB	0.5 μg/mL	-	BD Pharmingen (551033)
CD3	Mouse	HIT3a	FC	5 μL/10^6 cells	PE	Biolegend (300308)
CD4	Mouse	RPA-T4	FC	5 μL/10^6 cells	APC	Biolegend (300514)
CD45RA	Mouse	HI100	FC	5 μL/10^6 cells	APC	Biolegend (304111)
CD45RO	Mouse	UCHL1	FC	5 μL/10^6 cells	Brilliant Violet 421	Biolegend (304223)
CD62L	Mouse	DREG-56	FC	5 μL/10^6 cells	APC-CY7	Biolegend (304813)
CD69	Mouse	FN50	FC	5 μL/10^6 cells	APC	Invitrogen (MA1-10274)
CD8	Mouse	SK1	FC	5 μL/10^6 cells	BV421	Biolegend (344747)
JAG1	Goat	Polyclonal	WB	1:1000	-	R&D Systems (AF1277)
JAG1	Mouse	MHJ1-152	FC	2 μg/mL	PE	BD Pharmingen (565495)
PD-1	Mouse	NAT105	FC	5 μL/10^6 cells	Brilliant Violet 421	Biolegend (367421)

Table S3. Primer and probe sequences used in VCN-PCR titration protocol

Target		Primers (5′ to 3′ sequence)	Probes (5′ to 3′ sequence)
LTR	F	GCTAACTAGGGAACCCAC	CTTGCCTTGAGTGCTTCAAGTAGTG
	R	GCTAGAGATTTTCCACACTGA	
ALB	F	GCTGTGAAAAACCTCTGTTGG	AGTGGAAAATGATGAGATGCCTGCT
	R	GACATCCTTTGCCTCAGCAT	
